# Overview of systematic reviews of the effectiveness of reminders in improving healthcare professional behavior

**DOI:** 10.1186/2046-4053-1-36

**Published:** 2012-08-16

**Authors:** Amy Cheung, Michelle Weir, Alain Mayhew, Nicole Kozloff, Kaitlyn Brown, Jeremy Grimshaw

**Affiliations:** 1 Department of Psychiatry, University of Toronto, 33 Russell St., 3rd Floor Tower, Toronto, ON, Canada; 2 Cochrane Effective Practice and Organisation of Care Group Centre for Practice-Changing Research Ottawa Hospital Research Institute, 501 Smyth Road, Ottawa, ON, Canada; 3 Clinical Epidemiology Program Ottawa Hospital Research Institute Department of Medicine, University of Ottawa, 501 Smyth Road, Ottawa, ON, Canada

**Keywords:** Reminders, Professional behavior, Overview

## Abstract

**Objective:**

The purpose of this project was to conduct an overview of existing systematic reviews to evaluate the effectiveness of reminders in changing professional behavior in clinical settings.

**Materials and methods:**

Relevant systematic reviews of reminder interventions were identified through searches in MEDLINE, EMBASE, DARE and the Cochrane Library in conjunction with a larger project examining professional behavioral change interventions. Reviews were appraised using AMSTAR, a validated tool for assessing the quality of systematic reviews. As most reviews only reported vote counting, conclusions about effectiveness for each review were based on a count of positive studies. If available, we also report effect sizes. Conclusions were based on the findings from higher quality and current systematic reviews.

**Results:**

Thirty-five reviews were eligible for inclusion in this overview. Ten reviews examined the effectiveness of reminders generally, 5 reviews focused on specific health care settings, 14 reviews concentrated on specific behaviors and 6 reviews addressed specific patient populations. The quality of the reviews was variable (median = 3, range = 1 to 8). Seven reviews had AMSTAR scores >5 and were considered in detail. Five of these seven reviews demonstrated positive effects of reminders in changing provider behavior. Few reviews used quantitative pooling methods; in one high quality and current review, the overall observed effects were moderate with an absolute median improvement in performance of 4.2% (IQR: 0.5% to 6.6%).

**Discussion:**

The results support that modest improvements can occur with the use of reminders. The effect size is consistent with other interventions that have been used to improve professional behavior.

**Conclusion:**

Reminders appear effective in improving different clinical behaviors across a range of settings.

## Background

Reminders are a common approach to prompt clinicians to remember to perform critical tasks, such as monitoring of chronic conditions. Reminders have taken on many forms since their inception, evolving from simple paper reminders posted on medical charts to complex computerized reminders. While earlier versions were often more labor-intensive to administer, the increased use of electronic medical records (EMR) in clinical settings has made computerized reminders more inexpensive and feasible to implement.

There has been a parallel increase in the number of studies examining the effectiveness of reminders to improve clinical care delivered in different settings. The first study that examined the use of reminders was published in 1976 by MacDonald and demonstrated improvements in quality of care [1]. The first systematic review of reminders was published in 1987 by Haynes and included 135 studies [2]. Since that time, a multitude of primary studies and systematic reviews using different methods and approaches to examine the effectiveness of reminders for different disorders in diverse clinical settings have been published. Therefore, this overview attempts to summarize the literature and provide useful information to guide health care providers and administrators to more effectively use reminders in different clinical settings [3].

Overviews are a new approach to summarizing evidence, synthesizing results from multiple systematic reviews in a single, useful document ( http://www.cochrane-handbook.org/) [4]. This is particularly important in areas with overlapping reviews. Overviews identify high-quality, reliable systematic reviews and explore consistency of findings across reviews.

There have been two previous overviews on changing professional behavior in health care settings [5,6]; however, neither of them specifically explored the use of reminders. Therefore, this overview will examine the effectiveness of reminders in improving professional behavior in clinical settings using data from existing systematic reviews.

## Materials and methods

This overview was carried out in conjunction with the Rx for Change database ( http://www.rxforchange.ca). The database consists of quality-appraised and summarized systematic reviews on the effects of professional and other interventions on changing professional behavior that is regularly updated using sensitive searches of MEDLINE, EMBASE, DARE and the Cochrane Library [7]. For this overview, two individuals screened the titles and abstracts of systematic reviews in the Rx for Change database to identify relevant articles published before September 2009.

Ethics approval was not required for this overview. No formal protocol was drawn up for this review in advance.

According to the Cochrane Effective Practice and Organisation of Care (EPOC) group ( http://www.epoc.cochrane.org), reminders are defined as ‘patient or encounter specific information, provided verbally, on paper or on a computer screen, which is designed or intended to prompt a health professional to recall information.’ The population of interest was health professionals working in clinical settings. The intervention had to compare the effectiveness of reminders to other interventions or control. Only reviews that reported outcomes for professional performance (for example, prescribing, test ordering, patient education and so on) were included. Reviews that only examined knowledge of the professional as the outcome were excluded. Reviews primarily focused on reminders or reviews where studies assessing reminders could be clearly distinguished from studies on other interventions were included.

### Quality assessment

All eligible reviews were assessed independently by two individuals using the AMSTAR quality assessment tool (A Measurement Tool to Assess Systematic Reviews). AMSTAR is an 11-item tool to assess the methodological quality of systematic reviews that has been internally and externally validated and has been found to have good reliability [8,9].

### Data analysis

We conducted dual, independent data extraction on populations, interventions, comparisons and outcomes using a standardized form. Disagreements were resolved by consensus or consultation with a third individual. Only the latest version of updated reviews was included. Systematic reviews that were published in more than one source were treated as duplicate reviews with data extracted from the most comprehensive paper.

Within a review, studies were included in the analysis if they addressed reminder interventions, either as part of another component such as EMR or as their own entity. Given the limited data presented in many reviews, we used a vote-counting method to assess the effectiveness of the interventions [10]. We re-analyzed the results of each review by counting the proportion of positive studies reported regardless of statistical significance. We chose to focus on the direction of effect instead of the statistical significance because many of the included studies were cluster randomized trials with unit of analysis errors that do not reliably estimate the statistical significance of an intervention [11]. Unit of analysis errors are very common in cluster trials of professional behavior change interventions; Grimshaw *et al*. observed that approximately 50% of cluster randomized controlled trials (RCTs) on guideline dissemination and implementation strategies (including reminders) had unit of analysis errors [12]. Although it is theoretically possible to adjust for unit of analysis errors, cluster trials are rarely reported in sufficient detail to permit this. In addition, a number of these studies are small and may not be adequately powered for statistical significance.

Effectiveness in this overview was categorized as 1) generally effective (more than two-thirds of studies in a review demonstrated positive effects), 2) mixed effects (one-third to two-thirds of studies demonstrated positive effects) and 3) generally ineffective (fewer than one-third of studies demonstrated positive effects).

Summaries of the included reviews are reported in Additional file 1 along with the proportion of included studies that assessed reminder interventions in each systematic review. We also report the overall findings of each review as provided by the review authors as well as any quantitative analyses undertaken by the authors of the original reviews (see Additional file 1). The results of RCTs and studies examining multifaceted interventions versus reminder interventions alone are also presented.

Although all reviews are summarized and reported, we focused our conclusion on reviews of higher quality (AMSTAR >5) and more current (2003 or later).

Finally, we categorized the reviews into four groups for analysis based on their focus: 1) broad reviews (for example, on all types of reminders), 2) reviews of specific settings (for example, primary care), 3) reviews of specific behaviors (for example, prescribing), and 4) reviews of specific patient populations (for example, geriatric population).

## Results

**Figure 1:**
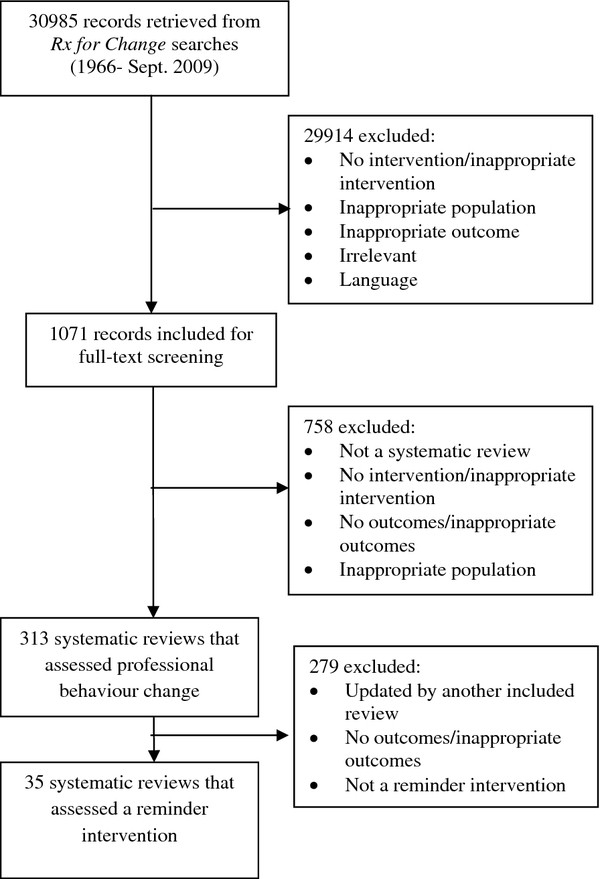
**Flow diagram of selected reviews.** Reviews included in the Rx for Change database that examined professional behavior change interventions.

**Figure 2:**
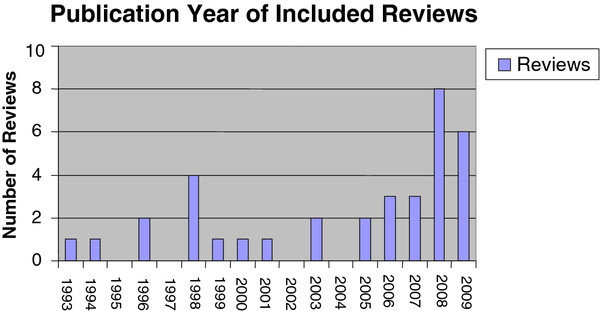
Publication year of included reviews.

There were 313 reviews included in the Rx for Change database that examined professional behavior change interventions (Figure 1), including 41 reviews of reminder interventions. We excluded six reviews because they had been updated by a subsequent review [2,13-17]. In total, 35 reviews published between 1993 and 2009 were eligible for inclusion in this overview. The majority of reviews were conducted from the late 1990s onward, and most broad reviews were published from the late 1990s to mid-2000s with fewer published in the last four years (Figure 2). 

Ten of the reviews looked at reminders generally, 5 reviews examined reminders in specific settings, 14 reviews looked at reminders for specific behaviors, and finally, 6 focused on reminders for specific patient populations.

**Figure 3:**
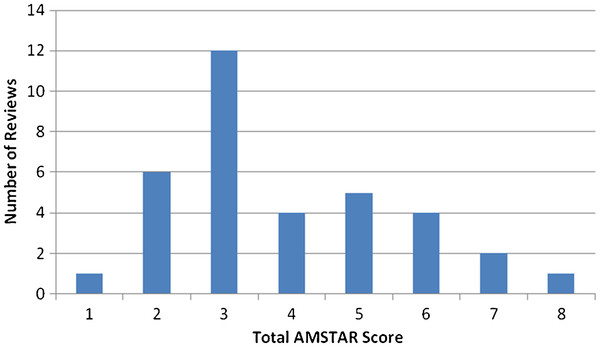
**AMSTAR scores of included reviews.** AMSTAR scores of included reviews.

The quality of the reviews was variable, the median AMSTAR score was 3 (range 1 to 8) (Figure 3 and Additional file 1). Several AMSTAR items were rarely reported in the included reviews: 1) working from a protocol (only reported by two reviews), 2) disclosing conflict of interest for individual studies (reported by no reviews), 3) assessing publication bias (two reviews), 4) searching grey literature (three reviews), and 5) listing included and excluded studies (three reviews). Further details on AMSTAR items are provided in Table 1.

**Table 1:** AMSTAR items

	**AMSTAR item**	**Number of reviews which met criteria (total 35)**
1	**Was an*a priori *design provided?**	2
2	**Was there duplicate study selection and data extraction?**	20
3	**Was a comprehensive literature search performed?**	26
4	**Was the status of publication (that is, grey literature) used as an inclusion criterion?**	3
5	**Was a list of studies (included and excluded) provided?**	3
6	**Were the characteristics of the included studies provided?**	27
7	**Was the scientific quality of the included studies assessed and documented?**	22
8	**Was the scientific quality of the included studies used appropriately in formulating conclusions?**	10
9	**Were the methods used to combine the findings of studies appropriate?**	21
10	**Was the likelihood of publication bias assessed?**	2
11	**Was the conflict of interest included?**	0

Data were extracted and analyzed from all 35 included reviews. However, only seven of the reviews had AMSTAR scores greater than 5. We focused our conclusions in the text below on these seven key reviews [17-23], but provide summaries of all included reviews in Additional file 1. We did not find any substantial discrepancies between the findings of the seven key reviews compared to the other identified reviews within the categories.

There was considerable overlap in the studies included in the systematic reviews. In total, 655 studies were included in the reviews, including 459 studies included in more than one review.

### Results from broad reviews

Out of the 10 reviews that broadly examined the effectiveness of reminders, including any health professionals in any clinical settings, half demonstrated that reminders were generally effective and half showed mixed results ( Additional file 1) [23-32].

Shojania *et al.* published the only high quality review (AMSTAR ≥8) in this category and demonstrated that reminders were effective [23]. The review included an analysis of 32 comparisons of on-screen computer reminders on process adherence, and was the only review that reported a quantitative summary for all included reminder studies based upon a description of the distribution (interquartile range and median) of the observed effects. The median effect size was an absolute risk difference of 4.2% (IQR: 0.5% to 6.6%) in the process of care measures. Shojania *et al.* also examined the impact of other effect modifiers on the effectiveness of computerized reminders and found that systems which required clinicians to provide a response were more likely to demonstrate a positive effect [23].

### Reviews of specific settings

A total of five reviews [33-37] examined studies that focused on specific health care settings, such as primary care or emergency rooms. Four of the five reviews showed positive results (three of five reviews: generally effective; one of five reviews: mixed results) ( Additional file 1). All of the settings evaluated were outpatient or ambulatory settings. These reviews were of lower quality: none of the reviews had an AMSTAR score >5.

### Reviews of specific behaviors

Fourteen of our included reviews focused on specific behaviors with the majority of studies examining prescribing changes [3,17,19,21,22,38-46]. All of the studies showed positive results (10/14 reviews: generally effective; 4/14 reviews: mixed results). Four of the reviews had AMSTAR scores >5 and all of these showed that reminders had a positive effect on professional behavior. Durieux *et al.* examined studies on the effect of computer-assisted drug dosing in a meta-analysis: the standardized mean difference (SMD) of initial doses favored the intervention and was statistically significant (five comparisons, SMD, 1.12; 95% confidence interval (C)], 0.33 to 1.92). There was a small non-significant pooled difference favoring the intervention in both maintenance dose changes (eight comparisons, SMD, 0.19; 95% CI, -0.10 to 0.48) and total amount of drug used (four comparisons, SMD, 0.43; 95% CI, -0.29 to 1.16) [19].

Ammenwerth and colleagues also found that reminders had positive effects on prescribing behaviors [17]. A subgroup analysis indicated that for locally developed systems (n = 12), the median effect was a 63% reduction in medication errors (range from reduction of 13% to a reduction of 99%) as compared to commercial systems (n = 11) with a median improvement of 47% reduction in medication errors (range from an increase of 26% to a reduction of 96%). Kaushal *et al.* did not restrict by profession and found that reminders were effective in improving prescribing [21]. Randell *et al*. evaluated the effect of reminders on nursing practice and the majority of studies eligible for this review demonstrated that the intervention was effective [22].

### Reviews of specific patient populations

The six remaining reviews focused on specific patient populations [18,21,47-50]. Five of the six reviews demonstrated that reminders were effective (four of six reviews: generally effective; one of six reviews: mixed results). Only two reviews scored >5 on the AMSTAR. Kastner *et al.* examined the effectiveness of reminders in improving management of osteoporosis and found mixed results [20]. Bywood and colleagues examined the use of reminders to improve management of drug and alcohol use disorders and found that reminders were generally ineffective in changing professional behavior [18].

## Discussion

In our overview of systematic reviews examining the effectiveness of reminders in improving professional behavior, we identified 35 systematic reviews with AMSTAR scores ranging from 1 to 8 (out of total score of 11) with a median of 3. The results of the reviews indicate positive effects when reminders are incorporated into a variety of clinical settings for different types of diseases. Furthermore, the results support that modest improvements can occur with the use of reminders, with one review estimating an overall effect size of 4.2%. This effect size is consistent with other interventions that have been used to improve professional behavior [12].

There are several strengths of this overview. First, it employed a comprehensive search strategy, developed and implemented by an information specialist as part of a larger project to examine interventions to change professional behavior. Second, duplicate screening, data extraction and quality assessments were conducted. Third, a validated instrument (AMSTAR) was used to assess the methodological quality of included reviews [8,9].

There are also several limitations to this overview. First, we did not retrieve data from the primary studies; therefore, we were limited by the information reported by the review authors on aspects such as the description of the interventions and outcomes. However, by focusing on the results of the systematic reviews rather than each individual primary study, we were able to obtain a broad sense of the field. We also focused our conclusions on reviews with AMSTAR scores >5 to address concerns with the quality of the systematic reviews. Second, this overview could not examine differences in effectiveness that may exist between locally developed and commercially available reminder systems due to the limited data. Only three of the included reviews evaluated the effectiveness of locally developed versus commercially available reminder systems [17,23,27]. The subgroup analysis conducted by Ammenwerth and colleagues demonstrated a higher relative risk reduction for locally developed systems, which they suggested was likely because they are developed to meet local needs, and sites often receive additional resources and support when implementing these systems [17]. Garg and colleagues also found that authors who created the decision support system were more likely to report improved performance [27], but this was not supported by Shojania *et al*. [23].

There may be other factors that impact the effectiveness of reminders, such as the functionality of the decision support system. Functionality (how well a system can model the clinician decision making process) was examined by two of the included reviews [20,24]. Both found that systems that actively engaged clinicians (either prompted them to use the system or required a response) improved performance to a greater degree compared with systems that required clinicians to initiate use.

Third, the 35 included systematic reviews are not independent given the significant overlap of the included studies. In total, 655 studies from the 35 reviews were included for analyses in this overview. One hundred and ninety-five studies were analyzed only once (found in only one included review) and 459 were “double-counted.” This overlap occurred most often with larger reviews that included more than 20 primary studies. In fact, 35% (160) of these studies were “double-counted” in the three largest systematic reviews in this overview [27,28,43]. Therefore, we would argue that these reviews should be considered as (partial) replications of systematic reviews of reminders undertaken by different authors with different inclusion criteria and methods. The convergence of findings across the reviews, therefore, is not surprising but reassuring that the findings are not due to the specific inclusion criteria or methods adopted by a group of authors.

Finally, there remains a lack of information on the long-term effect of reminders. None of the reviews restricted inclusion of studies based on length of follow-up, but the majority of studies were of relatively short durations. Whether the effectiveness of reminders diminishes over time has not been established to date.

An important methodological challenge in conducting overviews is how to interpret and summarize an area of research that includes reviews of variable quality. Another challenge is that inevitably, individual studies will be included in more than one systematic review, which leads to “double-counting.” To overcome both of these challenges, in this overview, we included all eligible reviews regardless of quality but we focused our conclusions on those of higher quality (AMSTAR >5). Therefore, we were able to provide a comprehensive picture of the state of the research literature on the effectiveness of reminders.

With 35 reviews included in this overview, and others still continuing to be published, evaluating the effectiveness of reminder interventions is a topic of frequent publication [51-54]. However, the literature is disorganized and reviews are often published in overlapping topic areas, which suggests that there is an unnecessary duplication of efforts by review authors [55]. Furthermore, the publication of reviews that focus on specific populations, settings or diseases (that is, split reviews) rather than broadly based reviews that include all professionals in all settings (that is, lumped reviews) also adds to the duplication of efforts since the former are really subgroup analyses of the latter. It is unclear whether further systematic reviews of reminders will likely change the conclusions of this overview, although we judge this unlikely, questioning the need for substantial new reviews in this area. Instead, we would argue that the field would be best served by updating (a limited number of) high quality broad reviews. Future reviews should focus on possible effect modifiers and moderators to explain the variation observed across primary studies of reminders. Given the poor quality of existing reviews, authors of future reviews must utilize more robust methods to conduct and report their reviews. This should lead to fewer but higher quality reviews, resulting in a more organized field of literature that is more interpretable by end-users.

## Conclusion

Reminders address acts of omission that are bound to occur because of simple overload of information for health care clinicians [1]. The results of this overview suggest that reminder systems are effective in changing healthcare professional behavior and improving processes of care. They may be more likely to be successful if they are designed to meet the specific needs of the clinical setting they are serving. Systems that proactively prompted clinicians and/or required a response were also more likely to be effective in changing professional behavior. Recent studies have also demonstrated the potential of checklists, a form of reminder, to dramatically improve patient morbidity and mortality [56]. Our findings suggest that the effects of reminders are positive and they may meaningfully impact clinical practice since they are relatively inexpensive and easy to administer in many settings, particularly as EMR becomes more common.

## Abbreviations

AMSTAR: A Measurement Tool to Assess Systematic Reviews; EPOC: Cochrane Effective Practice and Organisation of Care group; EMR: electronic medical records; RCTs: randomized controlled trials; SMD: standardized mean difference.

## Competing interests

Amy Cheung was supported by a Ministry of Health and Long-Term Care, Ontario, Career Scientist Award and the CIHR RCT Mentoring Program. Alain Mayhew is supported by the Canadian Institutes of Health Research and the Canadian Agency for Drugs and Technologies in Health. Michelle Weir was supported by the Canadian Agency for Drugs and Technologies in Health. Jeremy Grimshaw holds a Tier 1 Canada Research Chair. The website which provided the data, Rxforchange.ca is hosted by the Canadian Agency for Drugs and Technologies in Health. Amy Cheung (PI) had full access to all of the data in the study and takes responsibility for the integrity of the data and the accuracy of the data analysis. There are other competing interests.

## Authors’ contributions

JG, AM, MW and AC contributed to the design of the overview. AM, MW, AC, NK and KB conducted data extraction. All authors contributed to the analyses and completion of the manuscript. All authors read and approved the final manuscript.

## Supplementary Material

Additional file 1 **List and characteristics of included reviews.** Summaries of the included reviews are reported along with the proportion of included studies that assessed reminder interventions in each systematic review. Also reported are the overall findings of each review as provided by the review authors as well as any quantitative analyses undertaken by the authors of the original reviews. Click here for file
