# Supplementary material for: Overview of systematic reviews of the effectiveness of reminders in improving healthcare professional behavior
Source: Syst Rev. 2012 Aug 16;1:36. doi: 10.1186/2046-4053-1-36 (PMC3503870; doi:10.1186/2046-4053-1-36)
Supplement: Additional file 1 — List and characteristics of included reviews. Summaries of the included reviews are reported along with the proportion of included studies that assessed reminder interventions in each systematic review. Also reported are the overall findings of each review as provided by the review authors as well as any quantitative analyses undertaken by the authors of the original reviews. [file 2046-4053-1-36-S1.doc]

|  | **Broad** |  |  |  |  |  |  | |  |
| --- | --- | --- | --- | --- | --- | --- | --- | --- | --- |
|  | **Author/year** | **Focus** | **Quality score** | **Design of included studies (described in reviews)** | **# of included studies/**  **Search dates** | **Intervention/**  **comparisons** | **Main results** | | **Review Authors’ Conclusions** |
| 1 | Balas et al., 1996^24^ | **Type of Reminder:** Computerised  **Targeted Behaviour:**  All  **Professional Population:**  NS  **Patient Population:** NS | 1 | RCT | 98 papers included, 48 eligible for analyses,  (Search dates not available) | Reminders alone | 22/29 RCT favoured intervention | | Intervention was reported to demonstrate statistically significant improvement, but overall point estimates not provided.  Remaining studies did not assess reminder interventions |
|  |  |  |  |  |  | Reminders plus patient reminders | 19/19 RCT favoured intervention | |  |
| 2 | Buntinx et al., 1993^25^ | **Type of Reminder:** NS  **Targeted Behaviour:**  All  **Professional Population:**  MDs  **Patient Population:** NS | 2 | RCT, CCT | 26 studies included, 9 eligible for analyses,  Up to 1992 | Reminders alone | 7/9 favoured intervention  4/6 RCT favoured intervention | | No overall pooled estimate provided. Assume both paper and computerised reminders.  Seventeen studies in this review assessed the effect of feedback. |
| 3 | Chaudhry et al., 2006^26^ | **Type of Reminder:** Computerised  **Targeted Behaviour:**  NS  **Professional Population:**  NS  **Patient Population:** NS | 4 | RCT, BA, CCT, time series, qualitative studies, case control, cohort, | 257 studies included, 24 eligible for analyses,  1995-2004 | Reminders alone | 18/24 favoured intervention  12/17 RCT favoured intervention | | No overall pooled estimate provided.  Over 200 studies assessed other computerised interventions such as EMR. |
| 4 | Garg et al., 2005^27^ | **Type of Reminder:** CDSS  **Targeted Behaviour:**  All  **Professional Population:**  HCP  **Patient Population:** All | 5 | RCT, non-RCT | 100 studies included,  97 eligible for analyses,  1998-2004 | CDSS versus no CDSSs | 62/97 favoured intervention (number of RCTs not specified) | | Overall, 62/97studies demonstrated that CDSS improved practitioner performance.  3 studies did not assess practitioner performance  Found more positive effects if system developed locally.  Systems requiring a response from clinicians were more likely to have positive effects . |
| 5 | Kawamoto et al., 2006^28^ | **Type of Reminder:** CDSS  **Targeted Behaviour:**  NS  **Professional Population:**  All  **Patient Population:** NS | 5 | RCT | 70 studies, all RCTs, included with 71 comparisons included in the analysis,  1996-2003 | Reminders plus other | 48/71 comparisons favoured intervention (statistically significant) * no results available based on vote counting | | Overall effect showed that 48/70 studies favoured intervention.  Both paper and computerised reminders were included. |
| 6 | Mitchell and Sullivan, 2001^29^ | **Type of Reminder:** Computerised  **Targeted Behaviour:** NS  **Professional Population:**  NS  **Patient Population:** NS | 2 | RCT, CCT, CBA, case-series, **BA**, questionnaires and surveys | 89 studies included, 32 eligible for analyses,  Up to 1997 | Reminders alone | 20/25 favoured intervention  16/19 RCT favoured intervention | | No overall pooled estimate provided. Most studies did not include a reminder component |
|  |  |  |  |  |  | Reminders plus CPOE | 1/1 favoured intervention  (no results from RCT) | |  |
|  |  |  |  |  |  | Reminders plus other | 4/6 favoured intervention  3/4 RCT favoured intervention | |  |
| 7 | Nies et al., 2006^30^ | **Type of Reminder:** Computerised  **Targeted Behaviour:**  All  **Professional Population:**  NS  **Patient Population:** NS | 2 | RCT, non-RCT | 59 studies included, 20 eligible for inclusion (only summary vote counting results available),  Up to 2005 | Reminders plus CPOE | 12/20 favoured intervention (no results for RCT available) | | Overall, 31 of 59 studies were positive.  Thirty-nine studies either looked at patient or resource outcomes |
| 8 | Shiffman et al., 1999^31^ | **Type of Reminder:** Computerised  **Targeted Behaviour:**  NS  **Professional Population:**  NS  **Patient Population:** NS | 2 | RCT, CCT, time series | 20 studies included,  20 eligible for analyses,  1992-1998 | Reminders alone | 7/20 favoured intervention  4/9 RCT favoured intervention | | Authors report that 14/18 studies demonstrate an effect on some guideline adherence outcomes. |
| 9 | Shojania et al., 2009^23^ | **Type of Reminder:** Computerised  **Targeted Behaviour:**  All  **Professional Population: MDs**  **Patient Population:** NS | 8 | RCT, CCT | 28 studies included, 28 eligible for analyses,  Up to July 2008 | Reminders alone  8/8 favoured intervention | | 6/6 RCT favoured intervention | Overall effect size – median improvement of 4.2%.  Did not find difference between local versus commercially developed systems  If clinicians prompted to use the reminders system, effects were more likely to be positive.  Other effect modifiers showed no impact. |
|  |  |  |  |  |  | Reminders versus CPOE | | 5/10 favoured intervention  4/9 RCT favoured intervention |  |
|  |  |  |  |  |  | Reminders plus other | | 9/10 favoured intervention  7/7 RCT favoured intervention |  |
| 10 | Sintchenko et al., 2007^32^ | **Type of Reminder:** CDSS  **Targeted Behaviour:**  NS  **Professional Population:**  HCP  **Patient Population:** NS | 3 | RCT | 24 studies included, 14 eligible for analyses,  1994-2006 | Reminders alone | 7/9 RCT favoured intervention | | Overall, 13/24 of studies had positive result.  Ten studies did not clearly include reminders. |
|  |  |  |  |  |  | Reminders plus CPOE | 2/5 RCT favoured intervention | |  |

| **Specific Setting** | | | | | | | | |
| --- | --- | --- | --- | --- | --- | --- | --- | --- |
| 1 | Bryan et al., 2008^33^ | **Type of Reminder:** CDSS  **Targeted Behaviour:**  All  **Professional Population:**  Primary and ambulatory care workers  **Patient Population:** NS | 3 | RCT, observational, non-RCT | 17 studies included, 13 eligible for analyses,  1982-2006 | Specific Setting. | 7/13 favoured intervention  4/10 RCT favoured intervention | Overall 13/17 of studies had positive effect on some outcomes.  Four studies did not include reminders. |
| 2 | Colombet et al., 1999^34^ | **Type of Reminder:** Decision aids and reminders  **Targeted Behaviour:**  Triage patients with chest pain  **Professional Population:**  Emergency room staff  **Patient Population:** Emergency room patients with chest pain | 3 | RCT, timeseries | 11 studies included, 5 eligible for analyses,  Up to 1998 | Reminders only | 0/5 favoured intervention  0/2 RCT favoured intervention | According to authors, 7/11 studies favoured intervention.  Six studies did not include a reminder component. |
| 3 | Georgiou et al., 2007^35^ | **Type of Reminder:** Computerised with EMR  **Targeted Behaviour:**  NS  **Professional Population:**  NS  **Patient Population:** Outpatient setting | 3 | RCT, ITS, quasi experimental (lab-based), non RCT, BA | 19 studies included, 10 eligible for analyses,  1990-2004 | Reminders alone | 4/5 favoured intervention  2/3 RCT favoured intervention | No overall pooled estimate provided 9 studies did not include a reminder as part of the CPOE. |
|  |  |  |  |  |  | Reminders plus CPOE | 3/5 favoured intervention (no RCT included) |  |
| 4 | Jerant et al., 2000^36^ | **Type of Reminder:** Computerised  **Targeted Behaviour:**  All  **Professional Population:** Primary care MDs  **Patient Population:** Out patient setting | 3 | RCT, non-RCT | 16 studies included, 12 eligible for analyses,  Up to 1999 | Reminders alone | 7/10 favoured intervention  6/7 RCT favoured intervention | Overall 15 /16 studies reported positive results. 4 studies did not include a reminder as part of an EMR. |
|  |  |  |  |  |  | Reminders plus other | 2/2 RCT favoured intervention  Effect size not available |  |
| 5 | Shea et al., 1996^37^ | **Type of Reminder:** Computerised  **Targeted Behaviour:** Preventive care  **Professional Population:**  MD, patient  **Patient Population:** Ambulatory care, Prevention | 4 | RCT | 16 studies included, 10 eligible for analyses,  Up to 1994 | Reminders alone | 7/7 favoured intervention | OR ranges from 1.02 – 3.09 6 studies did not target professional behaviour |
|  |  |  |  |  |  | Reminders plus other | 2/3 favoured intervention |  |

| **Specific Behaviour** | | | | | | | | | | |
| --- | --- | --- | --- | --- | --- | --- | --- | --- | --- | --- |
| 1 | | Ammenwerth et al., 2008^17^ | **Type of Reminder:** Computerised  **Targeted Behaviour:** Prescribing  **Professional Population:**  MD  **Patient Population:** NS | 6 | RCT, time series, ITS, pre-post, cohort | 27 studies included, 20 eligible for analyses,  1990-2006 | Reminders plus CPOE | 19/20 favoured intervention  2/2 RCT favoured intervention | | 23/25 studies demonstrated an effect on medication errors.  Seven studies did not include a reminder with the electronic prescribing.  Found that locally developed systems had more favourable results.  Systems with advanced decision support also had more favourable effects. |
| 2 | | Austin et al., 1994^38^ | **Type of Reminder:** NS  **Targeted Behaviour:** Preventive care  **Professional Population:**  MD  **Patient Population:** NS | 2 | RCT | 4 studies included, 4 eligible for analyses,  Search dates not available | Reminders alone | 2/2 RCT favoured intervention | | OR ranges from 1.18 to 2.82. |
|  |  |  |  |  |  |  | Reminders plus other | 2/2 RCT favoured intervention | |  |
| 3 | Bennett et al., 2003^39^ | | **Type of Reminder:** Computerised, feedback  **Targeted Behaviour:** Medication management  **Professional Population:**  NS  **Patient Population:** NS | 4 | RCT | 26 studies included, 16 eligible for analyses,  1966-2001 | Reminders alone | | 8/16 RCT favoured intervention | Relative rate ranges from 1.0 to 42.0 based on all studies.  Seven studies evaluated effects of feedback and 3 addressed interventions for patient behaviour |
| 4 | Dexheimer et al., 2008^40^ | | **Type of Reminder:** Paper or computerised  **Targeted Behaviour:** Preventive care  **Professional Population:**  NS  **Patient Population:** NS | 3 | RCT | 28 new included (along with 30 from Balas),  1997-2004 (not including Balas search) | Reminders alone | | Individual study results not available in review, overall effect of 14% reported SD (12) | Effect size ranged from 10 to 14%.  Update of Balas and Weingarten, et al., 2000. |
|  |  |  |  |  |  |  | Reminders plus other | | Individual study results not available in review, overall effect of 10% reported SD (16) |  |
| 5 | Durieux 2008^19^ | | **Type of Reminder:** CADD  **Targeted Behaviour:**  Change in dosage  **Professional Population:**  HCP  **Patient Population:** NS | 7 | RCT, CCT, alternating time series design, | 23 included papers, 19 eligible for analyses,  1966-2006 | Reminders alone | 6/16 favoured intervention mixed designs: mixed effects 5/14 RCTs favoured intervention: mixed effects | | No overall estimate provided, SMD ranges from -0.55 to 1.12. Rate ratio for one comparison of 0.45 4 studies were not included because intervention was integrated into CPOE |
|  |  |  |  |  |  |  | Reminders plus CPOE | 2/3 mixed designs favoured intervention: generally effective  1/2 RCTs favoured intervention: mixed effects | |  |
| 6 | | Eslami et al., 2007^41^ | **Type of Reminder:** CPOE  **Targeted Behaviour:**  Safety, adherence to guidelines, and prescribing  **Professional Population:**  NS  **Patient Population:** NS | 2 | RCT, observational, non-RCT | 30 studies included, 11 eligible for analyses,  1950-2006 | Reminders plus CPOE | 6/11 favoured intervention  1/4 RCT favoured intervention | | No overall pooled estimate provided.  Nineteen studies, assessed CPOE without any reminder component  (reported by outcomes, not studies). |
| 7 | Jimbo et al., 2006^42^ | | **Type of Reminder:** Computerised  **Targeted Behaviour:** Prventive care (cancer)  **Professional Population:**  MD  **Patient Population:** NS | 3 | RCT, CBA, ITS, controlled trials | 30 studies included, 24 eligible for analyses,  1980-2005 | Reminders alone | 10/10 favoured intervention  6/6 RCT favoured intervention | | No overall pooled estimate provided.  Six studies assessed patient reminders for patient behaviour or the intervention did not include a reminder component |
|  |  |  |  |  |  |  | Reminders plus other | 12/14 favoured intervention  10/12 RCT favoured intervention | |  |

| 8 | Kaushal et al., 2003^21^ | **Type of Reminder:** CPOE and CDSS  **Targeted Behaviour:** Prescribing and dosing  **Professional Population:**  All  **Patient Population:** NS | 6 | RCT, retrospective/prospective BA, RCT cross-over, retrospective time series | 12 studies included, dates not specified. | Reminders alone  6/7 favoured intervention | 5/6 RCT favoured intervention | Overall, 9/12 studies favoured the intervention. |
| --- | --- | --- | --- | --- | --- | --- | --- | --- |
|  |  |  |  |  |  | Reminders plus CPOE | 5/5 favoured intervention  2/2 RCT favoured intervention |  |
| 9 | Pearson et al., 2009^43^ | **Type of Reminder:** CDSS  **Targeted Behaviour:** Prescribing  **Professional Population:**  NS  **Patient Population:** NS | 5 | RCT, quasi experimental | 56 studies included, 56 eligible for analyses,  1990-2007 | Reminders alone | 14/24 favoured intervention  9/19 RCT favoured intervention | 64 comparisons, within the 56 studies. 19/38 studies showed improvement in the majority of outcomes for initiating treatment; 8/23 showed improvement in the majority of outcomes for monitoring treatment; and 0/3 studies showed improvement in outcomes for stopping treatment |
|  |  |  |  |  |  | Reminders plus CPOE | 12/24 favoured intervention  11/23 RCT favoured intervention |  |
|  |  |  |  |  |  | Reminders plus other | 4/8 RCT favoured intervention |  |
| 10 | Randell et al., 2007^22^ | **Type of Reminder:** CDSS  **Targeted Behaviour:**  Nursing practice  **Professional Population:**  Nurses  **Patient Population:** NS | 6 | RCT, CCT, controlled BA, ITS | 8 studies included, 5 eligible for analyses,  Up to 2006 | Reminders alone | 4/5 RCT favoured intervention | Overall effect reported as “inconsistent”.  Remaining 3 studies only examined patient outcomes. |
| 11 | Reckmann et al., 2009^44^ | **Type of Reminder:** CPOE  **Targeted Behaviour:** Prescribing  **Professional Population:**  NS  **Patient Population:** NS | 3 | RCT, pre-post, time-series, retrospective and prospective cohort | 12 studies included, 12 eligible for analyses,  1950-2007 | Reminders plus CPOE r | 9/12 favoured intervention  1/1 RCT favoured intervention | All studies included in analysis and 9/12 demonstrated some improvement |
| 12 | Schedlbauer et al., 2009^45^ | **Type of Reminder:** Alerts  **Targeted Behaviour:** Prescribing  **Professional Population:**  HCP  **Patient Population:** NS | 5 | RCT, ITS, BA | 20 studies included, 19 eligible for analyses,  Up to May 2007 | Reminders plus CPOE | 17/19 favoured intervention  2/3 RCTs favoured intervention | 23 of 27 reminders in 20 studies demonstrated an improvement in behavior or decrease in errors.  One study did not include a reminder component. |
| 13 | Weir et al., 2009^3^ | **Type of Reminder:** CPOE and reminder  **Targeted Behaviour:** Prescribing  **Professional Population:**  MD  **Patient Population:** ICU patients | 5 | Pre-post, time series, case control | 46 studies included, 12 eligible for analyses,  1976 – mid 2007 | Reminders plus CPOE | 8/12 favoured intervention | No overall pooled estimate provided.  34 studies did not include a reminder component. |
| 14 | Yourman et al., 2008^46^ | **Type of Reminder:** CDSS  **Targeted Behaviour:** Prescribing  **Professional Population:**  NS  **Patient Population:** Older adults >= 60 | 3 | ITS, RCT, pre-post, cohort | 10 studies included, 9 eligible for analyses,  January 1980-July 2007 | Reminders alone | 5/6 favoured intervention  2/3 RCT favoured intervention | Overall, 8/10 studies showed modest improvement. |
|  |  |  |  |  |  | Reminders plus other | 3/3 favoured intervention  1/1 RCT favoured intervention |  |

| **Specific Patient Population** | | | | | | | | |
| --- | --- | --- | --- | --- | --- | --- | --- | --- |
| 1 | Bywood et al., 2008^18^ | **Type of Reminder:** Reminders and feedback  **Targeted Behaviour:**  All  **Professional Population:**  HCP  **Patient Population:** Patients with alcohol or drug issues | 6 | RCT, cohort, non RCT | 15 studies included, 12 eligible for analyses,  1966-2005 | Reminders alone | 2/12 favoured intervention  0/6 RCT favoured intervention | No overall pooled estimate provided.  Three studies focused on feedback. |
| 2 | Chatellier et al., 1998^47^ | **Type of Reminder:** Computerised  **Targeted Behaviour:** Prescribing  **Professional Population:**  NS  **Patient Population:** Patients receiving Anticoagulant Therapy | 3 | RCT | 7 studies eligible for analyses, 1966-1997 | Computer Assisted Prescribing System | 5/7 RCT favoured intervention | OR 1.29 for being in target range for anticoagulant therapy (95% CI 1.17-1.49). |
| 3 | Fitzmaurice et al., 1998^48^ | **Type of Reminder:** Computerised  **Targeted Behaviour:**  All  **Professional Population:** Physician  **Patient Population:**  Patients receiving Anticoagulant Therapy | 3 | RCT, other study design unclear | 7 studies included, results of only 4 provided in text, 1986-1995 | Reminders alone | 3/4 favoured intervention  1/1 RCT favoured intervention (first number includes all studies, second highlights the RCT findings included in the first value) | No overall pooled estimate provided.  Results not available for 3 of the 7 studies. |

| 4 | Kastner et al., 2008^20^ | **Type of Reminder:** CDSS  **Targeted Behaviour:**  Disease management  **Professional Population:**  HCP  **Patient Population:** Patients with Osteoporosis | 7 | RCT | 13 studies included, 10 eligible for analyses,  1966-2006 | Reminders alone | 1/2 RCT favoured intervention | No overall pooled estimate provided.  Three studies did not include a reminder component. |
| --- | --- | --- | --- | --- | --- | --- | --- | --- |
|  |  |  |  |  |  | Reminders plus other | 3/8 RCT favoured intervention |  |
| 5 | Montgomery et al., 1998^49^ | **Type of Reminder:** Computerised  **Targeted Behaviour:**  All  **Professional Population:**  NS  **Patient Population:**  Patients with hypertension | 4 | RCT | 6 studies included, 3 eligible for analyses, 1966-1997 | Reminders versus control | 2/3 RCT favoured intervention | No overall pooled estimate provided.  Three studies had no reminder component. |
| 6 | Van Rosse et al., 2009^50^ | **Type of Reminder:** Computerised  **Targeted Behaviour:** Prescribing  **Professional Population:** MDs  **Patient Population:**  ICU and Pediatrics patients | 3 | Controlled cross-sectional study, retrospective and prospective cohort | 12 studies included, 3 eligible for analyses up to 2007 | Reminders plus CPOE | 3/3 favoured intervention | For all comparisons, RR for medication prescribing errors was 0.08 (95% CI 0.01-0.770.  Nine studies did not include a reminder component. |

**Acronyms:**

BA: Before-after studies

CADD: Computer assisted drug dosage

CBA: Controlled before-after studies

CCT: Controlled clinical trials

CDSS: Computerised decision support system

CPOE: Computerised physician order entry

EMR: Electronic medical record

HCP: Health care professional

ICU: Intensive care unit

ITS: Interrupted time series study

MD: Medical doctor

NS: Not specified

RCT: Randomized controlled trial

SD: Standard deviation
